# Gender-Specific Differences in Sedation-Associated Outcomes During Complex Electrophysiological Procedures

**DOI:** 10.3390/healthcare13070844

**Published:** 2025-04-07

**Authors:** Lyuboslav Katov, Weronika Huggle, Yannick Teumer, Alexandra Buss, Federica Diofano, Carlo Bothner, Wolfgang Öchsner, Wolfgang Rottbauer, Karolina Weinmann-Emhardt

**Affiliations:** 1 Department of Cardiology, Ulm University Heart Center, Albert-Einstein-Allee 23, 89081 Ulm, Germany; lyuboslav.katov@uniklinik-ulm.de (L.K.); weronika.huggle@uni-ulm.de (W.H.); yannick.teumer@uniklinik-ulm.de (Y.T.); alexandra.buss@uniklinik-ulm.de (A.B.); federica.diofano@uniklinik-ulm.de (F.D.); carlo.bothner@uniklinik-ulm.de (C.B.); wolfgang.rottbauer@uniklinik-ulm.de (W.R.); 2 Department of Anesthesiology and Intensive Care Medicine, Ulm University Medical Center, Albert-Einstein-Allee 23, 89081 Ulm, Germany; wolfgang.oechsner@uniklinik-ulm.de

**Keywords:** deep sedation, electrophysiological procedures, gender-specific differences, venous blood gas analysis, hemodynamic outcomes, respiratory complications

## Abstract

**Background**: Interventional electrophysiology is a rapidly advancing field, with sedation essential for patient comfort and immobility during complex electrophysiological procedures (EPS). However, sedatives and analgesics can cause respiratory depression and hypotension. Gender-specific differences (GDs) are often overlooked in medical research, as protocols and dosages are typically based on male subjects, potentially compromising treatment safety and efficacy for women. This study examines GDs in CO_2_ levels, respiratory rate, arterial blood pressure (ABP), and anesthetic requirements during deep sedation for EPS. **Methods**: This prospective study at Ulm University Heart Center included 702 patients (405 men and 297 women) treated under deep sedation between August 2019 and October 2023. Standard monitoring included an electrocardiogram (ECG) with heart rate, non-invasive ABP, oxygen saturation (SpO_2_), and a frequent venous blood gas analysis (vBGA). The primary composite endpoint was GDs in SpO_2_ dips below 90% and pathological vBGA changes. **Results**: The primary composite endpoint was reached by 177 women (59.6%) and 213 men (52.6%), showing no significant difference (*p* = 0.102). Women had a 1,6-fold higher risk of experiencing SpO_2_ dips below 90% (*p* = 0.001). Additionally, women had 1.7 times higher hypoxia rates (*p* < 0.001) and were 1.5 times more likely to have a mean ABP below 65 mmHg (*p* < 0.001). On average, they received 65.3 mg less total propofol than men (*p* = 0.005) and a higher midazolam dose per kilogram of body weight (*p* < 0.001). **Conclusions**: Although the primary composite endpoint showed no significant GDs, secondary outcomes highlight the need to consider gender-specific sedation adjustments, particularly for women. This study underscores the need for personalized sedation management and patient monitoring regarding GDs.

## 1. Introduction

Catheter ablation is a widely used minimally invasive procedure for the treatment of supraventricular and ventricular cardiac arrhythmias [1,2]. In electrophysiological procedures, sedation plays a pivotal role in ensuring that patients remain immobile, experience adequate analgesia, and remain unconscious while also maintaining hemodynamic stability and optimal blood gas levels despite the respiratory depressive effects of sedation [3]. Effective sedation management is essential to minimize complications, such as hypoxia, hypercapnia, and hemodynamic instability, optimizing patient safety and procedural outcomes. Although there are already numerous studies on anesthesia in general, so far, a significantly higher number of men than women have been incorporated in randomized controlled trials (RCTs) on anesthesia-related interventions, and only 3% of these RCTs stratified their results by patient gender [4]. Nevertheless, these few studies indicate some differences in how men and women respond to anesthesia [5]. Studies suggest that women are more likely to experience anesthetic awareness and faster emergence from anesthesia and have higher bispectral index scores (BIS) at similar concentrations of anesthetics [6,7,8]. Additionally, women experience more pain than men after emerging from anesthesia, whereas men seem to experience more pain during deep sedation [8,9,10]. Furthermore, women often require higher amounts of sedation than men [11].

To summarize, although previous studies found differences between men and women concerning sedation, the whole field remains underexplored. Especially regarding gender-specific differences in vital parameters, such as peripheral oxygen saturation (SpO_2_) and peripheral blood gas levels during electrophysiological procedures (EPS), available data remain limited. Recognizing these differences could lead to a more gender-specific sedation approach, helping to prevent clinically significant deviations in vital parameters. This study aims to evaluate gender-specific differences during electrophysiological procedures under deep sedation.

## 2. Materials and Methods

### 2.1. Trial Design and Population

This prospective study included all patients scheduled for an electrophysiological examination under deep sedation, both male and female. Patients were enrolled from 20 August 2019 to 11 October 2023 at the Ulm University Heart Center. Eligible participants were individuals aged 18 years or older who required an electrophysiological intervention, such as cryo-pulmonary vein isolation or 3D-mapping-integrated atrial and ventricular interventions under deep sedation, and who provided written informed consent before the procedure. The exclusion criteria encompassed the absence of informed consent and interventions performed under mild sedation. The study protocol was approved by the Ethics Committee of the University of Ulm and adhered to the principles of the Declaration of Helsinki (protocol code 324/16, 12 October 2016).

### 2.2. Monitoring Setup and Sedation

Standard monitoring during the procedure included continuous electrocardiogram, heart rate, and pulse oximetric SpO_2_ measurements. Non-invasive blood pressure was measured every three minutes. A peripheral venous blood gas analysis was conducted at 30 min intervals. Transcutaneous CO_2_ levels were measured using a forehead electrochemical sensor in a subset of patients with the transcutaneous CO_2_ monitoring device (TCM400, Radiometer^®^, Brønshøj, Denmark) as part of the previously described block randomization [12].

Sedation was initiated with a 5 mg bolus of midazolam for anxiolysis, followed by a continuous propofol infusion to maintain deep sedation. Prior to ablation, fentanyl was administered for cryo-pulmonary vein isolation, whereas patients undergoing 3D-mapping-integrated interventions received a continuous remifentanil infusion. Airway patency was maintained using Wendel and/or Guedel tubes, with supplemental oxygen administered via a mask. A technical sedation assistant documented the sedation protocol, including the airway management interventions, peripheral venous blood gas results, and the timing and dosage of the administered drugs. During the study period, transesophageal echocardiography was not routinely performed as part of the procedure. It was primarily used pre-procedurally to exclude intracardiac thrombi in cases of insufficient oral anticoagulation and did not affect sedation management during the intervention.

### 2.3. Trial Endpoints

The primary composite endpoint comprised episodes of oxygen desaturation, defined as SpO_2_ levels falling below 90%, in combination with abnormal peripheral venous blood gas findings, including a partial pressure of CO_2_ (pCO_2_) exceeding 30% from baseline, a pCO_2_ level exceeding 70 mmHg, or a pH value decrease below 7.25. The secondary endpoint included the individual components of the primary composite endpoint, as well as a systolic blood pressure below 80 mmHg and a mean blood pressure below 65 mmHg. The secondary endpoint also considered variations in the administrated doses of sedatives and opioids, standardized as morphine equivalent doses.

### 2.4. Statistical Analysis

The statistical analysis was conducted using SPSS Statistics (Version 29, IBM, Armonk, NY, USA). The chi-square test was employed for categorical variables, whereas the Mann–Whitney U test was applied for continuous variables. Using logistic regression, the primary and secondary endpoints were calculated, and the differences between men and women were compared. Additionally, a separate logistic regression was conducted to evaluate the potential influence of TCM400 device usage between the groups. The anesthetic dosage used was compared through linear regression. Continuous variables were reported as median values along with interquartile ranges (IQR). A *p*-value below 0.05 was considered statistically significant.

## 3. Results

### 3.1. Patients’ Characteristics

A total of 726 patients were enrolled in the trial. However, 20 cases were excluded due to technical problems with the monitoring. An additional four cases were excluded from the statistical analysis due to lacking data. The study included 405 men (57.7%) and 297 women (42.3%). Among the male and female patients in the study, 306 underwent cryo-pulmonary vein isolation, 364 had a 3D-mapping-integrated atrial intervention, and 32 received VT ablation. The median (IQR) age of the patients was 68.0 (61.0; 76.0) years. On average, women were older than men (72.0 vs. 66.0 years; *p* < 0.001). A higher proportion of men had heart failure with reduced left ventricular function (51.7% vs. 31.4%; *p* < 0.001), coronary artery disease (43.7% vs. 26.3%; *p* < 0.001), as well as a higher prevalence of both past (22.5% vs. 11.0%; *p* < 0.001) and current (11.7% vs. 7.1%; *p* = 0.043) smoking. The remaining parameters showed no significant differences (Table 1).

### 3.2. Treatment Characteristics

The analysis of the treatment characteristics revealed that the median (IQR) procedure duration was 150.0 (110.0; 206.5) minutes. The baseline SpO_2_ was identical in both groups (*p* = 0.296), as was the initial O_2_ dose (8.0 (4.0; 8.0) L/min; *p* = 0.596). Lower SpO_2_ levels during the procedure were more frequently observed in women (*p* = 0.025). Although the mean blood pressure was lower in women (84.0 vs. 87.5 mmHg), the difference between the groups was not statistically significant (*p* = 0.233). A detailed summary of the treatment characteristics is provided in Table 2.

### 3.3. Primary Endpoint

**Figure 1:**
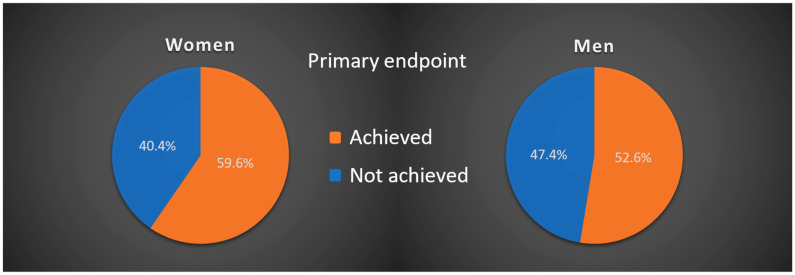
Depiction of primary endpoint outcomes in both groups. Patients who achieved the primary endpoint are represented in orange, while those who did not achieve it are shown in blue.

The composite primary endpoint, which included a decrease in SpO_2_ and changes in the venous blood gas analysis, was achieved by 55.6% (390 patients) of all treated patients. Among women, 59.6% (177 women) reached the primary endpoint, while it was 52.6% (213 men) among men. Overall, the comparison between the two genders showed no significant difference (*p* = 0.102) (Figure 1).

The presence of TCM400 CO_2_ monitoring had no impact on the occurrence of at least one primary endpoint in either group (*p* = 0.726; Odds Ratio 1.055).

To account for potential confounders, a multivariable logistic regression analysis was performed, adjusting for baseline characteristics, such as age, BMI, cardiovascular risk factors, and smoking history. No variable reached statistical significance; detailed results are available in Supplementary Table S1.

### 3.4. Secondary Endpoint

Analysis of the secondary endpoints revealed significant differences between male and female patients. Women had a 1.6-fold increased risk of experiencing a SpO_2_ drop below 90% during the procedure compared to men (*p* = 0.001). However, no significant differences were observed between genders in the risk of a pCO_2_ increase above 70 mmHg (*p* = 0.377) or a pCO_2_ rise exceeding 30% from baseline (*p* = 0.674). Similarly, the incidence of respiratory acidosis was comparable between men and women (*p* = 0.535). Women had a 1.5-fold higher risk of experiencing a mean arterial pressure drop below 65 mmHg compared to men (*p* < 0.001), whereas the occurrence of systolic blood pressure drops below 80 mmHg was similar between the sexes (*p* = 0.200). Additionally, hypoxia was significantly more frequent in female patients (*p* < 0.001). Differences were also observed in the administration of sedatives. There was no significant variation in the total midazolam dose administered between women and men (*p* = 0.966), whereas the midazolam dose per kilogram of body weight was higher in women (*p* < 0.001). Although all patients received a fixed 5 mg midazolam dose, the dose per kilogram body weight was higher in women due to their lower average body weight. On average, the total propofol dose was 65.3 mg lower in women (*p* = 0.005). However, the dose per kilogram of body weight showed no significant difference (*p* = 0.682). Similarly, the total doses of fentanyl (*p* = 0.022) and remifentanil (*p* = 0.005) were significantly lower in women, while no significant differences were observed when adjusted for body weight. A comprehensive overview of the endpoints is presented in Table 3. The subgroup analysis by ablation type revealed subtle differences in the sedation-related outcomes: SpO_2_ drops below 90% and hypoxia in women were more frequent in 3D-mapping and VT ablation procedures, while hypotension in women occurred more often during cryo-PVI and 3D-mapping interventions. Interestingly, in the VT ablation subgroup, no gender-specific difference was observed for hypotension despite a small sample size. Women received higher doses of midazolam per kilogram of body weight during cryo-PVI and 3D-mapping ablation procedures, with no significant differences observed in the administration of the other medications (Supplementary Tables S2–S4).

## 4. Discussion

Current research on gender-specific differences in deep sedation remains scarce. While previous studies have investigated variations in the anesthetic response between men and women, few have specifically examined the physiological impact of deep sedation during electrophysiological procedures [13,14,15,16]. Our study aims to bridge this gap by comparing vital parameters between male and female patients, with a primary focus on changes in the peripheral SpO_2_ and venous blood gas parameters. Additionally, we analyzed the hemodynamic responses, anesthetic requirements, and opioid administration patterns.

The primary composite endpoint, including SpO_2_ desaturations below 90% and pathological alterations in the peripheral venous blood gas analysis, was observed in more than half of the patients in both groups, with no significant difference between genders. Nonetheless, analysis of the secondary endpoints revealed significant gender-based differences, with women exhibiting a higher incidence of SpO_2_ drops below 90%, an increased prevalence of hypoxia, and a greater reduction in mean arterial blood pressure below 65 mmHg. These findings suggest that women may particularly benefit from enhanced monitoring strategies for early detection and management of such physiological disturbances while also indicating the potential need for adjustments in anesthetic dosing and ventilation parameters to better accommodate their specific physiological requirements.

The increased incidence of arterial hypotension in women contradicts some prior studies, which have reported that men are more prone to intraoperative hypotension, particularly in the initial 30 min following anesthesia induction [17]. Testosterone, along with other sex steroid hormones such as estrogen, plays a crucial role in modulating nitric oxide release, thereby influencing endothelial function [18]. Information on hormone replacement therapy was not routinely collected during the study period, which limits the ability to account for its potential impact on sedation outcomes. Additionally, circulating catecholamine levels are essential for maintaining optimal hemodynamics during and after the procedure, contributing to rapid stabilization and cardiovascular adaptation [19,20,21]. While men tend to have higher blood pressure at younger ages, this trend shifts with aging. By the age of 65 to 70 years, hypertension becomes more prevalent in women than in men, with rates continuing to rise as women age [20]. Moreover, women may experience a prolonged post-interventional recovery period due to pharmacodynamic drug effects, with progesterone and estrogen contributing to this phenomenon [8]. The aforementioned factors underscore the intricate interplay of hormonal influences on vascular tone and autonomic regulation, highlighting the need for further investigation.

Another notable finding in the current trial is the gender-based difference in opioid administration, with women receiving significantly higher total doses of fentanyl than men. This aligns with previous research indicating that women exhibit higher cytochrome P450 3A4 (CYP3A4) activity, leading to an accelerated metabolism of fentanyl and necessitating higher doses to achieve the desired analgesic effect [11,22]. Additionally, studies have demonstrated that women tend to exhibit a heightened pain response compared to men, potentially contributing to increased opioid requirements [23]. However, this effect appears to be specific to fentanyl and does not necessarily extend to other opioids, such as remifentanil, as demonstrated in our study [24]. The variability in opioid pharmacokinetics and receptor sensitivity between genders underscores the need for personalized analgesic regimens based on individual metabolic and physiological characteristics. Furthermore, it remains unclear whether the respiratory depressant effects of fentanyl could have contributed to the increased incidence of hypoxia and other respiratory complications observed in female patients. Given that women required lower total sedative doses overall, it is conceivable that opioid-induced respiratory depression played a more pronounced role in this group. In addition to pharmacokinetic and metabolic differences, psychophysiological factors such as pain perception and anxiety may also contribute to the observed gender disparity in opioid requirements. Women have been shown to exhibit lower pain thresholds and higher pain sensitivity compared to men, which may lead to greater analgesic needs under procedural sedation [23,25]. Moreover, elevated baseline anxiety levels, which are more prevalent among female patients, could increase the sedative and opioid requirements and potentially exacerbate respiratory or hemodynamic instability [26]. These findings emphasize the importance of tailoring opioid titration strategies to minimize the risk of respiratory compromise in female patients.

An unexpected observation was the higher prevalence of pathological respiratory parameters in women, despite the fact that men in our study had a higher prevalence of smoking, coronary artery disease, and heart failure with reduced left ventricular ejection fraction. Conventional expectations would suggest that male patients, due to their greater burden of cardiopulmonary comorbidities, would be at a higher risk for respiratory complications [27]. This observation raises the important question of whether women, despite having fewer underlying conditions, inherently exhibit reduced physiological resilience to deep sedation. Possible explanations for this discrepancy may include differences in lung function, ventilatory drive, hormonal modulation of respiration, and differential susceptibility to sedative-induced respiratory depression. Furthermore, women have been shown to have higher baseline respiratory rates and lower functional residual capacity, which may predispose them to more rapid oxygen desaturation during periods of apnea or hypoventilation [28]. Due to their lower average body weight, cardiac output, and reduced liver blood flow, females potentially metabolize sedatives and opioids more slowly, which could result in prolonged respiratory depression [29]. Additionally, progesterone has been found to influence respiratory control, potentially contributing to gender-based differences in ventilatory responses to sedation [30,31].

Obesity, chronic respiratory conditions, and cardiovascular disease are well-established factors that influence physiological responses to deep sedation [32,33]. In our cohort, approximately one-third of patients were classified as obese, highlighting the clinical relevance of this comorbidity. Excess body weight is associated with reduced lung compliance, increased airway resistance, and impaired diaphragmatic excursion, all of which may promote hypoventilation and gas exchange disturbances under sedation [34]. Similarly, conditions such as obstructive sleep apnea, COPD, and cardiovascular disease can affect baseline oxygenation, vascular reactivity, and autonomic regulation, thereby altering the response to sedation agents [35,36]. Notably, sex-specific differences in the prevalence and pathophysiology of these comorbidities may partially explain the divergent sedation-related outcomes observed between male and female patients [37,38]. To reduce potential confounding, we adjusted the endpoint analyses for body mass index (BMI) and age—two key variables known to impact respiratory and hemodynamic stability during sedation [39]. These adjustments enhance the robustness of our gender-based comparisons, though further studies incorporating a broader range of covariates are warranted so as to fully disentangle the biological sex effects from comorbidity-related influences.

The findings in this study highlight the need for further research to explore gender-specific physiological responses to anesthesia and sedation. Understanding these differences could facilitate the development of individualized anesthesia management protocols, incorporating tailored ventilatory strategies, modified drug dosing, and optimized perioperative monitoring for both male and female patients. Given the increased incidence of hypoxia, hypotension, and altered opioid metabolism in women, anesthesia protocols should account for gender-based physiological differences to enhance patient safety.

## 5. Conclusions

In conclusion, no significant differences between men and women regarding the primary combined endpoint were found. However, significant gender-specific differences were observed in the frequency of SpO_2_ dips, hypoxia, and mean arterial blood pressure. The results of this study underscore the need for personalized, gender-specific sedation management and patient monitoring. Large-scale randomized studies are needed to further evaluate this important topic.

## Figures and Tables

**Table 1:** Patients’ characteristics.

	Total (*n* = 702)	Women (*n* = 297)	Men (*n* = 405)	*p*-Value
Age [years], median (IQR)	68.0 (61.0; 76.0)	72.0 (63.0; 78.0)	66.0 (58.0; 74.0)	<0.001
BMI, median (IQR)	27.6 (24.5; 31.1)	27.6 (23.7; 31.5)	27.5 (24.8; 30.9)	0.414
Obesity, *n* (%)	222.0 (31.7)	99.0 (33.4)	123.0 (30.4)	0.399
Reduced LVEF, *n* (%)	305.0 (43.1)	94.0 (31.4)	211.0 (51.7)	<0.001
Arterial hypertension, *n* (%)	520.0 (74.1)	225.0 (75.8)	295.0 (72.8)	0.383
Coronary artery disease, *n* (%)	255.0 (36.3)	78.0 (26.3)	177.0 (43.7)	<0.001
Hyperlipoproteinemia, *n* (%)	431.0 (61.5)	181.0 (60.9)	250.0 (61.9)	0.801
Diabetes mellitus, *n* (%)	117.0 (16.7)	47.0 (15.8)	70.0 (17.3)	0.608
Smoker, *n* (%)	68.0 (9.7)	21.0 (7.1)	47.0 (11.7)	0.043
Former smoker, *n* (%)	121.0 (17.6)	32.0 (11.0)	89.0 (22.5)	<0.001
COPD, *n* (%)	41.0 (5.8)	18.0 (6.1)	23.0 (5.7)	0.831
Asthma, *n* (%)	22.0 (3.1)	13.0 (4.4)	9.0 (2.2)	0.105
OSAS, *n* (%)	49.0 (7.0)	15.0 (5.1)	34.0 (8.4)	0.088
Pulmonary hypertension, *n* (%)	21.0 (3.0)	8.0 (2.7)	13.0 (3.2)	0.695

BMI, body mass index; COPD, chronic obstructive pulmonary disease; IQR, interquartile range; LVEF, left ventricular ejection fraction; OSAS, obstructive sleep apnea syndrome.

**Table 2:** Procedural characteristics.

	Total (*n* = 702)	Women (*n* = 297)	Men (*n* = 405)	*p*-Value
Procedure time (min), median (IQR)	150.0 (110.0; 206.5)	143.0 (110.0; 195.0)	157.0 (113.0; 212.0)	0.062
Cardioversion during procedure, *n* (%)	346.0 (48.2)	150.0 (49.3)	196.0 (47.1)	0.908
Defibrillation during procedure, *n* (%)	18.0 (2.4)	4.0 (1.3)	14.0 (3.2)	0.449
Initial O_2_ flow (L/min), median (IQR)	8.0 (4.0; 8.0)	8.0 (4.0; 8.0)	8.0 (4.0; 8.0)	0.596
Baseline SpO_2_ (%), median (IQR)	98.0 (97.0; 99.0)	99.0 (97.0; 100.0)	98.0 (97.0; 99.0)	0.296
Lowest recorded SpO_2_ (%), median (IQR)	87.0 (77.0; 93.0)	85.0 (74.0; 92.0)	88.0 (77.0; 93.0)	0.025
Baseline heart rate (bpm), median (IQR)	77.0 (62.0; 94.0)	77.0 (64.0; 96.0)	76.0 (62.0; 91.0)	0.154
Baseline sys. blood pressure (mmHg), median (IQR)	115.0 (101.5; 129.0)	115.0 (100.0; 135.0)	115.0 (102.0; 127.0)	0.418
Baseline mean blood pressure (mmHg), median (IQR)	86.0 (76.0; 96.0)	84.0 (75.0; 97.0)	87.5 (77.0; 95.5)	0.233

Bp, blood pressure; Bpm, beats per minute; IQR, interquartile range; min, minutes; O_2_, oxygen; SpO_2_, peripheral capillary oxygen saturation; sys., systolic.

**Table 3:** Overview of the endpoints between both groups.

	Total (*n* = 702)	Women (*n* = 297)	Men (*n* = 405)	*p*-Value	Regression Coefficient B *
SpO_2_ level < 90%, *n* (%)	308.0 (49.2)	151.0 (56.8)	157.0 (43.6)	0.001	0.483
Venous pCO_2_ > 70 mmHg, *n* (%)	29.0 (4.2)	10.0 (3.4)	19.0 (4.8)	0.377	−0.129
Increase of venous pCO_2_ > 30% from baseline, *n* (%)	125.0 (18.1)	51.0 (17.3)	74.0 (18.6)	0.674	0.072
Venous pH < 7.25, *n* (%)	125.0 (18.1)	50.0 (17.0)	75.0 (18.8)	0.535	−0.181
Systolic bp < 80 mmHg, *n* (%)	276.0 (40.2)	124.0 (43.1)	152.0 (38.2)	0.200	0.093
Mean bp < 65 mmHg, *n* (%)	371.0 (54.1)	177.0 (61.5)	194.0 (48.7)	<0.001	0.436
Hypoxia, *n* (%)	278.0 (44.4)	139.0 (52.3)	139.0 (38.6)	<0.001	0.499
Midazolam (mg), median (IQR)	5.0 (5.0; 5.0)	5.0 (5.0; 5.0)	5.0 (5.0; 5.0)	0.966	0.075
Midazolam (mg/kg), median (IQR)	0.06 (0.05; 0.07)	0.07 (0.06; 0.08)	0.06 (0.05; 0.06)	<0.001	0.011
Propofol (mg), median (IQR)	570.0 (370.0; 800.0)	500.0 (330.0; 797.0)	600.0 (399.0; 835.0)	0.005	−65.333
Propofol (mg/kg), median (IQR)	6.8 (4.6; 9.7)	6.9 (4.7; 9.7)	6.8 (4.5; 9.7)	0.682	0.421
Morphin equivalent dose Fentanyl i.v. total (mg), median (IQR)	0.0 (0.0; 16.5)	0.0 (0.0, 16.5)	0.0 (0.0; 16.5)	0.022	2.688
Morphin equivalent dose Fentanyl i.v. (mg/kg), median (IQR)	0.3 (0.2; 0.4)	0.3 (0.2; 0.4)	0.3 (0.2; 0.4)	0.159	0.036
Morphin equivalent dose Remifentanil i.v. total (mg), median (IQR)	3.2 (0.0; 6.9)	0.0 (0.0; 6.6)	3.6 (0.0; 7.5)	0.005	−2.979
Morphin equivalent dose Remifentanil i.v. (mg/kg), median (IQR)	0.08 (0.06; 0.11)	0.08 (0.06; 0.11)	0.08 (0.06; 0.11)	0.564	−0.030

* BMI- and age-adjusted. Bp, blood pressure; IQR, interquartile range; i.v., intravenous; kg, kilogram of body weight; pCO_2_, partial pressure of carbon dioxide; pH, potential of hydrogen; SpO_2_, peripheral capillary oxygen saturation.

## Data Availability

The data presented in this study are available on request from the authors. The data are not publicly available due to data privacy laws.

## Supplementary Materials

The following supporting information can be downloaded at: https://www.mdpi.com/article/10.3390/healthcare13070844/s1, Table S1: Results of the multivariable binary logistic regression analysis evaluating predictors of the primary endpoint, Table S2: Overview of the endpoints between both groups in the cryo-PVI group, Table S3: Overview of the endpoints between both groups in the 3D-mapping LA ablation group, Table S4: Overview of the endpoints between both groups in the 3D-mapping VT ablation group.

## Abbreviations

The following abbreviations are used in this manuscript:

ABP	Arterial blood pressure
BIS	Bispectral index scores
BMI	Body mass index
BP	Blood pressure
BPM	Beats per minute
CO_2_	Carbon dioxide
COPD	Chronic obstructive pulmonary disease
CYP3A4	Cytochrome P450 3A4
Df	Degrees of freedom
EPS	Electrophysiological procedures
Exp(B)	Exponentiated coefficient
GDs	Gender-specific differences
IQR	Interquartile range
LA	Left atrial
LVEF	Left ventricular ejection fraction
OSAS	Obstructive sleep apnea syndrome
pCO_2_	Partial pressure of carbon dioxide
pH	Potential of hydrogen
pO_2_	Partial pressure of oxygen
RCTs	Randomized controlled trials
SpO_2_	Peripheral oxygen saturation
TCM	Transcutaneous CO_2_ monitoring
vBGA	Venous blood gas analysis
VT	Ventricular tachycardia
