# Supplementary material for: Gender-Specific Differences in Sedation-Associated Outcomes During Complex Electrophysiological Procedures
Source: Healthcare (Basel). 2025 Apr 7;13(7):844. doi: 10.3390/healthcare13070844 (PMC11988496; doi:10.3390/healthcare13070844)
Supplement: Supplementary file 1 [file healthcare-13-00844-s001.zip › healthcare-3556305-supplementary.pdf]

Supplemental Table S1. Results of the multivariable binary logistic regression analysis evaluating predictors of the primary endpoint

	Regression coefficient B	Standard Error	Wald	df	p-value	Exp (B)
Women	-0.325	0.168	3.749	1	0.053	0.723
Age at intervention	-0.002	0.008	0.081	1	0.776	0.998
BMI	0.012	0.014	0.691	1	0.406	1.012
Coronary artery disease	0.100	0.185	0.291	1	0.589	1.105
Arterial hypertension	-0.051	0.199	0.065	1	0.799	0.951
Hyperlipoproteinemia	-0.239	0.170	1.990	1	0.158	0.787
Diabetes mellitus	0.364	0.220	2.735	1	0.098	1.439
Nicotine abuse	0.286	0.280	1.043	1	0.307	1.331
Former nicotine abuse	-0.228	0.219	1.088	1	0.297	0.796
Constant	-0.119	0.864	0.019	1	0.891	0.888

BMI, body mass index; Df, degrees of freedom; Exp(B), exponentiated coefficient.

Supplemental Table S2. Overview of the endpoints between both groups in the cryo-PVI group

	Total *(n=306)*	Women *(n=148)*	Men *(n=158)*	p-value
Primary endpoint achieved, n (%)	153.0 (50.0)	81.0 (54.7)	72.0 (45.6)	0.109
SpO2 level <90%, n (%)	142.0 (50.2)	76.0 (55.9)	66.0 (44.9)	0.065
Venous pCO2 >70 mmHg, n (%)	5.0 (1.7)	3.0 (2.1)	2.0 (1.3)	0.584
Increase of venous pCO2 >30% from baseline, n (%)	37.0 (12.3)	19.0 (13.1)	18.0 (11.5)	0.664
Venous pH <7.25, n (%)	29.0 (9.6)	14.0 (9.7)	15.0 (9.6)	0.976
Systolic bp <80 mmHg, n (%)	109.0 (36.1)	61.0 (41.8)	48.0 (30.8)	0.046
Mean bp <65 mmHg, n (%)	145.0 (48.0)	80.0 (54.8)	65.0 (41.7)	0.022
Hypoxia, n (%)	129.0 (45.6)	70.0 (51.5)	59.0 (40.1)	0.056
Midazolam (mg), median (IQR)	5.0 (5.0; 5.0)	5.0 (5.0; 5.0)	5.0 (5.0; 5.0)	0.840
Midazolam (mg/KG), median (IQR)	0.06 (0.05; 0.07)	0.06 (0.06; 0.08)	0.05 (0.05; 0.06)	<0.001
Propofol (mg), median (IQR)	458.5 (310.0; 682.5)	430.0 (300.0; 607.5)	500.0 (320.0; 700.0)	0.089
Propofol (mg/KG), median (IQR)	5.6 (3.9; 8.1)	5.7 (4.1; 8.3)	5.3 (3.8; 7.8)	0.252
Morphin equivalent dose Fentanyl i.v. total (mg), median (IQR)	16.5 (16.5; 33.0)	16.5 (16.5; 33.0)	33.0 (16.5; 33.0)	0.262
Morphin equivalent dose Fentanyl i.v. (mg/KG), median (IQR)	0.3 (0.2; 0.4)	0.3 (0.2; 0.4)	0.3 (0.2; 0.4)	0.247
Morphin equivalent dose Remifentanil i.v. total (mg), median (IQR)	0	0	0	N/A
Morphin equivalent dose Remifentanil i.v. (mg/KG), median (IQR)	0.07 (0.04; 0.10)	0.07 (0.05; 0.11)	0.07 (0.04; 0.11)	0.756

Bp, blood pressure; IQR, interquartile range; i.v., intravenous; KG, kilogram of body weight; N/A, not applicable; pCO_2, partial pressure of carbon dioxide; pH, potential of hydrogen; SpO_2, peripheral capillary oxygen saturation; PVI, pulmonary vein isolation.

Supplemental Table S3. Overview of the endpoints between both groups in the 3D-mapping LA ablation group

	Total (*n=364)*	Women (*n=142)*	Men (*n=222)*	p-value
Primary endpoint achieved, n (%)	209.0 (57.4)	89.0 (62.7)	120.0 (54.1)	0.105
SpO2 level <90%, n (%)	147.0 (46.8)	68.0 (55.3)	79.0 (41.4)	0.016
Venous pCO2 >70 mmHg, n (%)	13.0 (3.6)	2.0 (1.4)	11.0 (5.1)	0.068
Increase of venous pCO2 >30% from baseline, n (%)	72.0 (20.1)	27.0 (19.0)	45.0 (20.8)	0.674
Venous pH <7.25, n (%)	85.0 (23.7)	33.0 (23.2)	52.0 (24.1)	0.856
Systolic bp <80 mmHg, n (%)	143.0 (40.6)	59.0 (43.7)	84.0 (38.7)	0.354
Mean bp <65 mmHg, n (%)	202.0 (57.4)	91.0 (67.4)	111.0 (51.2)	0.003
Hypoxia, n (%)	131.0 (36.0)	62.0 (50.4)	69.0 (36.1)	0.012
Midazolam (mg), median (IQR)	5.0 (5.0; 5.0)	5.0 (5.0; 5.0)	5.0 (5.0; 5.0)	0.601
Midazolam (mg/KG), median (IQR)	0.06 (0.05; 0.07)	0.07 (0.05; 0.08)	0.06 (0.05; 0.06)	<0.001
Propofol (mg), median (IQR)	680.0 (440.0; 997.5)	645.0 (400.0; 1000.0)	688.0 (450.0; 992.5)	0.400
Propofol (mg/KG), median (IQR)	7.9 (5.3; 11.0)	8.2 (5.7; 12.0)	7.7 (5.1; 10.6)	0.255
Morphin equivalent dose Fentanyl i.v. total (mg), median (IQR)	0	0	0	N/A
Morphin equivalent dose Fentanyl i.v. (mg/KG), median (IQR)	0.2 (0.2; 0.2)	0	0.2 (0.2; 0.2)	N/A
Morphin equivalent dose Remifentanil i.v. total (mg), median (IQR)	6.6 (4.3; 8.6)	6.6 (4.0; 7.9)	6.6 (4.3; 8.6)	0.182
Morphin equivalent dose Remifentanil i.v. (mg/KG), median (IQR)	0.08 (0.06; 0.11)	0.09 (0.06; 0.11)	0.08 (0.06; 0.11)	0.516

Bp, blood pressure; IQR, interquartile range; i.v., intravenous; KG, kilogram of body weight; LA, left atrial; N/A, not applicable; pCO_2, partial pressure of carbon dioxide; pH, potential of hydrogen; SpO_2, peripheral capillary oxygen saturation.

Supplemental Table S4. Overview of the endpoints between both groups in the 3D-mapping VT ablation group

	Total *(n=32)*	Women *(n=7)*	Men *(n=25)*	p-value
Primary endpoint achieved, n (%)	28.0 (87.5)	7.0 (100.0)	21.0 (84.0)	0.258
SpO2 level <90%, n (%)	19.0 (65.5)	7.0 (100.0)	12.0 (54.5)	0.028
Venous pCO2 >70 mmHg, n (%)	11.0 (34.4)	5.0 (71.4)	6.0 (24.0)	0.020
Increase of venous pCO2 >30% from baseline, n (%)	16.0 (50.0)	5.0 (71.4)	11.0 (44.0)	0.200
Venous pH <7.25, n (%)	11.0 (34.4)	3.0 (42.9)	8.0 (32.0)	0.593
Systolic bp <80 mmHg, n (%)	24.0 (75.0)	4.0 (57.1)	20.0 (80.0)	0.217
Mean bp <65 mmHg, n (%)	24.0 (75.0)	6.0 (85.7)	18.0 (72.0)	0.459
Hypoxia, n (%)	18.0 (62.1)	7.0 (100.0)	11.0 (50.0)	0.018
Midazolam (mg), median (IQR)	5.0 (5.0; 5.0)	5.0 (5.0; 7.5)	5.0 (5.0; 5.0)	0.324
Midazolam (mg/KG), median (IQR)	0.06 (0.05; 0.07)	0.07 (0.06; 0.11)	0.06 (0.04; 0.07)	0.148
Propofol (mg), median (IQR)	797.0 (467.5; 1237.5)	794.0 (350.0; 1400.0)	800.0 (510.0; 1175.0)	0.562
Propofol (mg/KG), median (IQR)	10.0 (6.2; 14.5)	8.5 (6.5; 15.7)	10.4 (5.7; 14.3)	1.000
Morphin equivalent dose Fentanyl i.v. total (mg), median (IQR)	0	0	0	N/A
Morphin equivalent dose Fentanyl i.v. (mg/KG), median (IQR)	0	0	0	N/A
Morphin equivalent dose Remifentanil i.v. total (mg), median (IQR)	7.9 (4.1; 11.7)	7.9 (3.3; 13.2)	7.9 (4.9; 10.6)	0.824
Morphin equivalent dose Remifentanil i.v. (mg/KG), median (IQR)	0.11 (0.07; 0.14)	0.12 (0.07; 0.41)	0.10 (0.07; 0.13)	0.251

Bp, blood pressure; IQR, interquartile range; i.v., intravenous; KG, kilogram of body weight; N/A, not applicable; pCO_2, partial pressure of carbon dioxide; pH, potential of hydrogen; SpO_2, peripheral capillary oxygen saturation; VT, ventricular tachycardia.
